# Percutaneous computed tomography-guided cryoablation for recurrent retroperitoneal soft tissue sarcoma: a study of safety and efficacy

**DOI:** 10.18632/oncotarget.9476

**Published:** 2016-05-19

**Authors:** Wenzhe Fan, Lizhi Niu, Yu Wang, Yingqiang Zhang, Xuehua Yao, Guosheng Tan, Jianyong Yang, Jiaping Li

**Affiliations:** ^1^ Department of Interventional Oncology, The First Affiliated Hospital of Sun Yat-Sen University, Guangzhou, China; ^2^ Fuda Cancer Hospital, Guangzhou, China; ^3^ Fuda Institute of Cryosurgery for Cancer, Guangzhou, China; ^4^ Department of Interventional Radiology, The First Affiliated Hospital of Sun Yat-Sen University, Guangzhou, China; ^5^ Department of Medical Imaging, The First Affiliated Hospital of Sun Yat-Sen University, Guangzhou, China

**Keywords:** computer tomography, cryoablation, retroperitoneal sarcoma, survival, response rate

## Abstract

**Aims:**

To evaluate the use of computed tomography image-guided percutaneous cryoablation for recurrent retroperitoneal soft tissue sarcomas (RPSs).

**Results:**

Adverse events were limited to grades 1 and 2, included fever (n = 19), local pain (n = 11), emesis (n = 10), frostbite (n = 6), and nerve injury (n = 1). Fever was more frequent in the large tumor group (15.8%) than in small tumor group (1.9%) (*P* = 0.008). Median PFS and OS were 37.0 ± 7.7 months (range, 4–39 months) and 43.0 ± 5.9 months (range, 6–54 months), respectively. PFS and OS were significantly longer in the small tumor group than in the large tumor group (*P* = 0.011 and *P* = 0.015, respectively), but the response rate (82.7% vs. 72.8%, *P* = 0.240) did not differ significantly. On univariate analysis, tumor size, tumor invasion grade, and distant metastasis were significant prognostic factors for PFS and OS. On multivariate analysis, a tumor size ≥10 cm was an independent negative prognostic factor for PFS and OS after cryoablation (HR: 3.98, 95% CI: 1.27–12.50, *P* = 0.018 and HR: 4.33, 95% CI: 1.41–13.26, *P* = 0.010, respectively).

**Materials and Methods:**

Data from 72 patients with recurrent RPSs who underwent percutaneous cryoablation were reviewed retrospectively. The prognostic factors for progression-free survival (PFS), overall survival (OS), and efficacy based on mRECIST criteria were analysis. Adverse events were compared according to tumor size (<10 and ≥10 cm).

**Conclusion:**

Minimally invasive percutaneous cryoablation was safe and efficacious for recurrent RPSs.

## INTRODUCTION

Retroperitoneal soft tissue sarcomas (RPSs) are neoplasms that originate in the retroperitoneum but not in the major retroperitoneal organs themselves. They account for approximately 0.15% of all malignancies [1]. These tumors are particularly difficult to manage because of their proximity to vital structures in the abdominal cavity and adjacent compartments [2]. Surgery is the primary, and potentially only, curative therapy for localized disease, and the overall reported 5-year survival rates are in the range of 60% [3, 4]. However, challenges associated with RPS resection may affect survival and lead to local recurrence. Tumor re-excision is recommended in case of RPS recurrence; however, repeat resection is even more challenging, and whether adjuvant chemotherapy and radiotherapy offer additional benefits is controversial [5].

Local ablative therapies such as radiofrequency ablation, microwave ablation, and cryoablation are established modalities in the palliative treatment of solid tumors [6–9]. Cryoablation in particular, with its advantages of inducing tumor necrosis by ice-ball formation, good visible ice ball in computed tomography (CT) image, and alleviation of the visceral cancer pain of abdomen, has been widely used to successfully treat abdominal neoplasms [6–9]. Percutaneous cryoablation can prolong survival in patients with advanced-stage or recurrent tumors, including hepatocellular and pancreatic cancers [6, 8].

The clinical benefit of palliative percutaneous cryoablation in patients with recurrent RPSs has not yet been investigated. In this study, the safety, efficacy, adverse events, local effects, and long-term outcomes were analyzed retrospectively to evaluate percutaneous cryoablation for recurrent RPSs by using CT guidance. The advantages of concomitant CT include rapid imaging, clear indication of spinal involvement, and good visualization of the ice-ball during cryoablation [10–12]. We hypothesized that more favorable outcomes would be produced by combining these two techniques.

## RESULTS

### Clinical data

A total of 72 consecutive patients were included, with a median age of 49.0 years (range, 25–86 years) (Table 1). Among them, spinal invasion was detected in 4 patients, renal invasion in 3, and ureteral invasion in 2. A total of 94 tumors were treated by cryoablation, including a single tumor in 56 patients, 2 tumors in 10, and 3 tumors in 6. A total of 166 cryoablation procedures were performed: 3 patients were treated with 4 cryoablations, 27 with 3 cryoablations, 33 with 2 cryoablations, and 7 with a single procedure. Of these cases, 52 cryoablations were performed in the small tumor group and 114 in the large tumor group. Distant metastases were found in 6 patients, including 3 in the retroperitoneal lymph nodes, 1 in the lung, 1 in the liver, and 1 in the iliac bone.

**Table 1:** Comparison of the clinicopathologic and demographic features between patients in the small and large tumor groups

Factor	Total n = 72	Small tumor group (<10 cm), n = 28	Large tumor group (≥10 cm), n = 44	*P*-value
**Sex**				0.625
Male	29 (40.3%)	10 (35.7%)	19 (43.2%)	
Female	43 (59.7%)	18 (64.3%)	25 (56.8%)	
**Age (years)**	49.0 ± 14.1	49.8 ± 14.6	48.5 ± 14.1	0.454
<50	49 (68.1%)	21 (75.0%)	28 (63.6%)	0.438
≥50	23 (31.9%)	7 (25.0%)	16 (36.4%)	
**Pathology**				0.467
Liposarcoma	38 (52.8%)	15 (53.6%)	23 (52.3%)	
Fibrosarcoma	19 (26.4%)	9 (32.1%)	10 (22.7%)	
Leiomyosarcoma	15 (20.8%)	4 (14.3%)	11 (25.0%)	
**FNCLCC grade**				0.084
I	14 (19.4%)	8 (28.6%)	6 (13.6%)	
II	37 (51.4%)	13 (46.4%)	24 (54.5%)	
III	21 (29.2%)	7 (25.0%)	14 (31.8%)	
**Tumor size before resection**	12.9 ± 4.2	13.2 ± 3.7	12.8 ± 4.6	0.723
**Time after resection (months)**	40.5 ± 38.5	47.2 ± 41.4	35.9 ± 36.5	0.347
**KPS score**	78.9 ± 9.4	80.8 ± 10.0	77.5 ± 8.9	0.250
**Invasion**				0.081
Absence	63 (87.5%)	27 (96.4%)	36 (81.8%)	
Presence	9 (12.5%)	1 (3.6%)	8 (18.1%)	
**Tumor number**				0.252
Single	56 (77.8%)	24 (85.7%)	32 (72.7%)	
≥2	16 (22.2%)	4 (14.3%)	12 (27.3%)	
**Distant metastasis**				0.394
Absence	66 (91.7%)	27 (96.4%)	39 (88.6%)	
Presence	6 (8.3%)	1 (3.6%)	5 (11.4%)	

Data are expressed as mean ± SD or number (percentage). Abbreviation: KPS, Karnofsky performance status

### Adverse events

All adverse events observed after cryoablation were mild to moderate grade 1–2 events including fever, emesis, skin frostbite, and local pain. All adverse events resolved with or without symptomatic treatment within 2 weeks. After 166 total cryoablations, the number of patients experiencing fever, local pain, emesis, skin frostbite, and nerve injury were 19 (11.4%), 11 (6.6%), 10 (6.0%), 6 (3.6%), and 1 (0.6%), respectively. The rate of fever in the large tumor group was significantly higher than that in the small tumor group (15.8% vs. 1.9%, *P* = 0.008). The frequency of procedure-related local pain and emesis were not significantly different between the small versus large tumor groups (5.8% vs. 7.0% and 1.9% vs. 7.9%, respectively, *P* > 0.05 for both groups). In the large tumor group, local superficial partial-thickness frostbite was apparent near the skin puncture site, and the fifth right-sided lumbar spine nerve root was injured in 1 case (Table 2). The nerve injury resulted in numbness and grade 2 muscle weakness of the right lower limb, which was relieved after 13 days of dehydration treatment using intravenous injection of mannitol.

**Table 2:** Adverse events in patients with recurrent RPS after each cryoablation

	Fever (n = 19)	Local pain (n = 11)	Emesis (n = 10)	Skin frostbite (n = 6)	Nerve injury (n=1)
**Grade**					
1 (Mild)	11	10	7	0	0
2 (Moderate)	8	1	3	6^*^	1
**Major tumor size (post cryoablation)**					
<10 cm	1 (1.9%)	3 (5.8%)	1 (1.9%)	0	0
≥10 cm	18 (15.8%)	8 (7.0%)	9 (7.9%)	6 (5.3%)	1 (0.9%)
*P*-value	0.008	1.000	0.174	N/A	N/A

* skin frostbite was not mentioned in Common Terminology Criteria for Adverse Events v4.0. However, local superficial partial-thickness skin frostbite was identified in this study, which was local and noninvasive. Abbreviation: N/A, not applicable

### Local effects

In the small tumor group, 6 cryoablation procedures (11.5%) achieved complete response (CR) and 37 (71.2%) achieved partial response (PR) according to the modified Response Evaluation Criteria in Solid Tumors (mRECIST), resulting in an objective response rate (RR) of 82.7%. The viable tumor size was stable in 6 patients (11.5%) but increased in 3 patients (5.8%).

**Figure 1:**
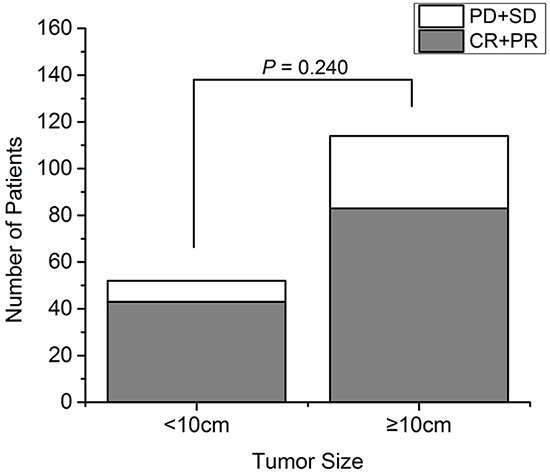
The response rate of 72 patients with recurrent RPS analyzed according to tumor size The response rate after cryoablation of small and large tumors (χ^2^ test, *P* = 0.240). CR: complete response; PD: progressive disease; PR: partial response; SD: stable disease

In the large tumor group, 83 cryoablations (72.8%) achieved PR but no CR was observed, resulting in an objective RR of 72.8%. The viable tumor size was stable in 18 patients (15.8%) but increased in 13 patients (11.4%). The total overall RR was 75.9%, and did not differ significantly between the two groups (*P* = 0.240; Figure 1).

### Progression-free survival and overall survival

**Figure 2:**
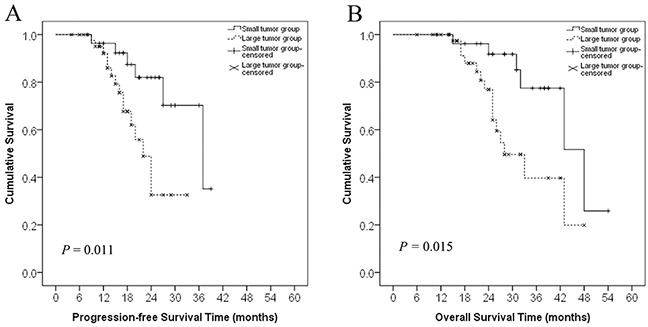
Progression-free survival and overall survival curves in patients with recurrent RPS in the small and large tumor groups **A.** Progression-free survival curves of patients in the small (<10 cm) and large (≥10 cm) tumor groups. The difference between the groups was statistically significant (log-rank test, *P* = 0.011). **B.** Overall survival curves of patients in the small and large tumor groups. The difference between the groups was statistically significant (log-rank test, *P* = 0.015).

The median progression-free survival (PFS) and overall survival (OS) rates were 37.0 ± 7.7 months (range, 4–39 months) and 43.0 ± 5.9 months (range, 6–54 months), respectively. As of June 30, 2015, 21 patients had died (6 in the small tumor group and 15 in the large tumor group). The overall PFS rates at 1, 2, and 3 years were 80.6%, 23.6%, and 4.2%, respectively. In the small tumor group, the 1-, 2-, and 3-year PFS rates were 89.3%, 39.3%, and 10.7%, respectively; in the large tumor group, these rates were 75.0%, 13.6%, and 0%, respectively. The PFS rates were significantly poorer for patients in the large tumor group compared to those in the small tumor group (log-rank test, *P* = 0.011, Figure 2A). Although the RR was similar between the small and large tumor group, the duration of PFS was significantly different between groups.

The overall 1-, 2-, and 3-year OS rates were 94.4%, 58.3%, and 18.1%, respectively. In the small tumor group, the 1-, 2-, and 3-year OS rates were 96.4%, 78.6%, and 32.1%, respectively; these rates in the large tumor group were 93.2%, 45.5%, and 9.1%, respectively. The OS rates were significantly poorer for patients in the large tumor group than those in the small tumor group (log-rank test, *P* = 0.015, Figure 2B).

Univariate analysis was undertaken to evaluate the impact of various factors, including sex, age, pathology, The French National Federation of the Centers for the Fight Against Cancer (FNCLCC) grade, tumor invasion, tumor size, tumor number, and distant metastasis on PFS and OS. Tumor size, tumor invasion FNCLCC grade, and distant metastasis were significant prognostic factors for PFS and OS. Multivariate analysis showed that having a tumor ≥10 cm alone was an independent negative prognostic factor for PFS and OS after cryoablation, with a HR of 3.98 (95% confidence interval [CI]: 1.27–12.50, *P* = 0.018) and HR of 4.33 (95% CI: 1.41–13.26, *P* = 0.010), respectively (Tables 3 and 4).

**Table 3:** Univariate and multivariate analyses of progression-free survival in 72 patients with recurrent RPS undergoing cryoablation

Factor	Univariate			Multivariate		
	HR	95% CI	*P*-value	HR	95% CI	*P*-value
**Sex**						
Female	1.00					
Male	1.19	0.51–2.82	0.687	0.92	0.35–2.37	0.856
**Age (years)**						
<50	1.00					
≥50	0.99	0.39–2.50	0.987	1.16	0.41–3.27	0.786
**Pathology**						
Liposarcoma	1.00					
Fibrosarcoma	1.64	0.46–5.83	0.446	1.06	0.33–3.45	0.924
Leiomyosarcoma	1.49	0.36–6.26	0.568	0.53	0.14–1.97	0.339
**FNCLCC grade**						
I	1.00					
II	1.78	0.59–5.34	0.304	1.85	0.53–6.39	0.333
III	6.41	1.42–28.94	0.016	6.71	0.69–65.31	0.101
**Invasion**						
Absence	1.00					
Presence	13.31	2.55–69.42	0.002	2.84	0.20–39.61	0.438
**Tumor size**						
<10 cm	1.00					
≥10 cm	3.45	1.24–9.61	0.011	3.98	1.27–12.50	0.018
**Tumor number**						
Single	1.00					
≥2	2.04	0.66–6.28	0.214	1.20	0.29–5.01	0.799
**Distant metastasis**						
Absence	1.00					
Presence	9.01	1.61–50.41	0.012	0.81	0.73–8.98	0.864

**Table 4:** Univariate and multivariate analyses of overall survival in 72 patients with recurrent RPS undergoing cryoablation

Factor	Univariate			Multivariate		
	HR	95% CI	*P*-value	HR	95% CI	*P*-value
**Sex**						
Female	1.00					
Male	1.22	0.51–2.89	0.654	1.06	0.38–2.97	0.910
**Age** (years)						
<50	1.00					
≥50	0.89	0.35–2.24	0.805	0.87	0.28–2.73	0.814
**Pathology**						
Liposarcoma	1.00					
Fibrosarcoma	2.01	0.57–7.06	0.279	0.78	0.24–2.48	0.669
Leiomyosarcoma	1.67	0.39–7.23	0.491	0.33	0.08–1.43	0.138
**FNCLCC grade**						
I	1.00					
II	2.70	0.73–9.98	0.136	2.07	0.47–9.07	0.336
III	16.60	2.83–97.28	0.002	21.37	2.31–97.34	0.070
**Invasion**						
Absence	1.00					
Presence	11.70	3.55–29.73	<0.001	27.23	1.91–89.00	0.055
**Tumor size**						
<10 cm	1.00					
≥10 cm	3.03	1.16–7.90	0.023	4.33	1.41–13.26	0.01
**Tumor number**						
Single	1.00					
≥2	2.63	0.84–8.27	0.099	3.07	0.61–15.34	0.173
**Distant metastasis**						
Absence	1.00					
Presence	10.24	2.58–55.21	<0.001	11.25	1.67–19.22	0.957

## DISCUSSION

RPSs represent approximately 80–85% of all primary retroperitoneal tumors, and can occur at any age and with similar frequency between men and women. They are characterized by local recurrence in 70% of cases within 10 years [13, 14]. Data on the tolerability and efficacy of cryoablation as a palliative treatment for recurrent RPS are lacking. In the present study, 72 patients with 94 malignant RPS recurrences underwent 166 cryoablations under local anesthesia. Cryoablation for RPSs was safe and well-tolerated, most likely because the tumors were minimally invasive and thus allowed for accurate targeting of the tumor volume.

Fever was the most frequent adverse event, especially in those with larger tumors. This suggests that having larger tumors may increase the risk of ablation-related infection and stronger antitumor immunity but the mechanism behind these possibilities requires further investigation [8–11]. Although more frequent skin frostbite and a single case of nerve injury were seen in the large tumor group, the numbers are too small to make any definitive statement. In addition to the ice-ball's proximity to the skin, frostbite may also be exacerbated by the number of probes used as well as the ice-ball melting duration [12]. Moreover, other factors such as vascularity and patient body mass index may affect frostbite risk. Local edema, which invariably occurs after freezing, might compress the local nerves [7]. However, the advantage of perioperative local anesthesia lies in the ability of patients to move their limbs and therefore avoid neurological impairments. Hence, only transient spinal nerve injuries were observed in one patient after a single cryoablation session.

Previous studies indicated that, as with surgical resection, complete local control for patients with aggressive tumors was difficult to achieve with cryoablation. Havez et al. [15] used cryoablation to treat 17 patients with extra-abdominal desmoid tumors and demonstrated a 100% objective RR, while *in situ* tumor recurrences were noted in 2 patients. In the present study, the total RR was 75.9% after 166 cryoablations, and although there were no differences in RR on the basis of tumor size, a CR was only observed in tumors <10 cm. The PFS and OS of patients with tumors ≥10 cm were significantly poorer than those of patients with tumors <10 cm, possibly because of insufficient coverage of the tumor or imprecise placement of the cryoprobes in large lesions [16]. Because large tumors are more likely to be situated close to adjacent organs, a safety margin of ice ball margin plus 5-10mm is necessary. The extent of this safety margin has been associated with an increased risk of inadequate treatment and local progression. To induce complete necrosis in smaller tumors, the ice-ball should extend at least 3–5 mm beyond the lesion periphery [12]; it is impossible to achieve these parameters using a single cryoablation session in tumors >10 cm.

Current American Joint Committee on Cancer (AJCC) soft tissue sarcoma (STS) staging is based on data examining prognostic factors in patients with STS lesions in their extremities; however, because of essential differences in disease characteristics, the applicability of these data to RPS staging is limited [17, 18]. The FNCLCC system might be more appropriate to predict distant metastasis development and tumor mortality in STS [19], as it was devised using the most important determinants of survival in localized STSs, such as cellular differentiation, mitotic rate, and tumor necrosis. We utilized this staging system, and as expected, we found that FNCLCC grade III RPS was significantly associated with poor survival, but only on univariate analysis.

Metastasis and local invasion are proven prognostic factors associated with decreased survival rates in patients with RPS [20–22]. Univariate analysis showed that both of these factors were associated with unfavorable PFS and OS in patients with RPS treated by cryoablation in our study; however, their significances were not evident upon multivariate analysis. Because a possible selection bias in the present study may have attributed to the fact that patients receiving cryoablation were less likely to have local invasion or distant metastases, which could have skewed the results. Multicenter trials are necessary to confirm the results obtained from retrospective studies such as ours.

The data obtained at our institution regarding the management of recurrent RPS by cryoablation compared well with other published data in terms of the risk factors associated with PFS and OS following surgical resection of primary tumors [23, 24]. However, our sample size was small, especially in the small tumor group, which may cause interpretation bias. Furthermore, the mean follow-up duration was only 45 months, which may underestimate the survival time of the patients and cause an inaccurate assessment of survival factors.

In conclusion, percutaneous cryoablation appeared to be a safe and effective treatment for recurrent RPSs with minimal invasion or without invasion. Tumor size, tumor invasion FNCLCC grade, and distant metastasis were significant prognostic factors for PFS and OS. A tumor size ≥10 cm was an independent negative prognostic factor for PFS and OS. Further studies in a more homogeneous population with longer follow-up periods are required to validate the clinical benefit of cryoablation on the survival of patients with RPS.

## MATERIALS AND METHODS

### Ethics statement

The protocol of this retrospective study was approved by our Institutional Review Board. Written informed consent was obtained from patients prior to treatment.

### Patients

Between January 2008 and June 2015, 222 patients with a diagnosis of recurrent RPS detected by biopsy or CT scanning and magnetic resonance imaging (MRI) were treated comprehensively after surgical resection at our institution. Patients were ineligible for further resection because of multiple tumors, aggressive local tumors in vital organs such as the kidney, ureters, abdominal aorta, and vertebral column, or because of refusal to undergo surgery. One-hundred and forty-four patients were excluded because they received other treatments (resection [n = 85], chemotherapy [n = 23], radiation [n = 19], and thermal ablation [n = 17]). A total of 78 patients underwent cryoablation as first-line anticancer treatment. Among them, 6 patients were excluded for reason that included resection (n = 3), post cryoablation radiotherapy (n = 2), and loss to follow-up (n = 1). Ultimately, 72 patients were included in the analyses. Treated tumors consisted of 3 histologic types including liposarcomas, fibrosarcomas, and leiomyosarcomas. The FNCLCC criteria were used for tumor grading (grades I, II, and III) [25].

### Data recording

Clinical and laboratory data were collected from all patients prior to cryoablation. Clinical data included age, sex, symptoms, Karnofsky performance score (KPS, 0 for dead and 100 for no complaints of disease) [26], tumor resection history, and pathology. Additionally, data on abdominal CT or MRI findings including tumor size (maximum dimension), number, and adjacent organ invasion were collected. The local recurrence rate was higher in patients with dermatofibrosarcoma whose tumors were ≥10 cm as described in a recent study [12]. Therefore, patients were divided into 2 groups based on the maximum tumor diameter before cryoablation: a small tumor group (<10 cm, n = 28) and a large tumor group (≥10 cm, n = 44).

### Cryoablation procedure

**Figure 3:**
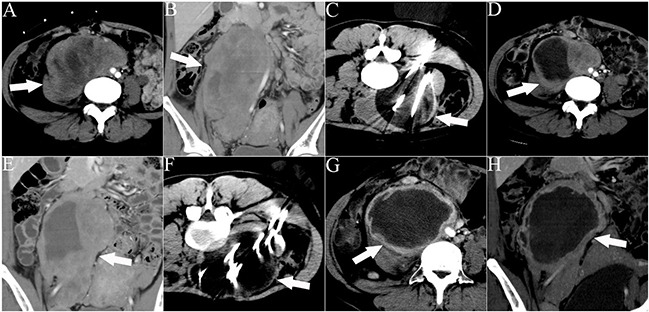
CT images of a 32-year-old woman who underwent 2 sessions of cryoablation for a recurrent RPS The patient experienced abdominal distension and pain for 1 month. The white arrows indicate the lesion. The white lines indicate the cryoprobes. The dark area around the cryoprobe is the ice-ball. **A, B.** Cross-sectional and coronal contrast-enhanced CT images illustrating the lesion with an abdominal aorta push. **C.** The first session of cryoablation using 11 cryoprobes. **D, E.** Cross-sectional and coronal contrast-enhanced CT images acquired 1 month after the first session of cryoablation illustrating necrosis of the lesion and lesion shrinkage. **F.** The second session of cryoablation using 10 cryoprobes. **G, H.** Cross-sectional and coronal contrast-enhanced CT images acquired 1 month after the second cryoablation session illustrating significant necrosis of the lesion and lesion shrinkage.

**Figure 4:**
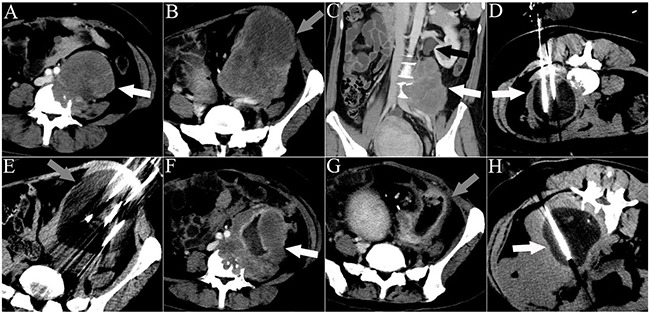
CT images of a 33-year-old man who underwent 3 sessions of cryoablation for 2 RPSs The patient presented with abdominal pain for 45 days. The white arrowheads indicate lesion 1. The gray arrowheads indicate lesion 2. The black arrowhead indicates ureteral dilation. The white lines indicate the cryoprobes. The dark area around the cryoprobe is the ice-ball. **A.** A cross-sectional contrast-enhanced CT image illustrating lesion 1 with spinal invasion. **B.** A cross-sectional contrast-enhanced CT image illustrating lesion 2. **C.** A coronal contrast-enhanced CT image illustrating lesion 1 with ureter invasion. **D.** The first session of cryoablation for lesion 1. **E.** The second session of cryoablation for lesion 2, performed 1 month after the first session. **F, G.** Cross-sectional contrast-enhanced CT images acquired 1 month after the second session of cryoablation illustrate necrosis of both lesions and significant shrinkage of lesion 2. **H.** The third session of cryoablation for lesion 1.

CT-guided procedures were performed by experienced interventional radiologists (WZF, LZL, YW, JYY, and JPL with 6, 15, 14, 30, and 20 years of experience, respectively), and post-cryoablation examinations were performed with a CT scanning system (Toshiba Xpress/SX, Tokyo, Japan). CT examinations were performed with contrast material (iopromide, Ultravist 300; Bayer HealthCare Pharmaceuticals) before cryoprobe insertion and after ablation. Intermittent CT scanning (120 kVp, 100 mA) was used for cryoprobe placement. Cryoablations were performed using an argon gas-based cryosurgical system and a 17-G cryoprobe (IceRod^TM^; Endocare, Irvine, CA) generating a 2-cm-wide and 4-cm-long ice-ball per probe. After local anesthesia via application of 1% lidocaine (Jincheng HEALTH Pharmaceutical Ltd., China) to the skin puncture sites, the probes were placed under CT guidance (contrast-enhanced and 3D volume reconstruction) 1–1.5 cm apart (Figures 3 and 4). The number of cryoprobes used, arranged as regular prism and polyhedron models with overlap as previously described by Chen et al. [27], was determined by size, geometry, and tumor morphology to adequately cover the lesion with the resulting ice-ball. Since cell death is certain only within 3 mm of the ice-ball margin where a temperature of −20°C can be attained, a 5- to 10-mm safety margin is necessary when the tumor is adjacent to vital organs (such as the spine, vessel, kidney, or intestine). Otherwise, complete ablation can be achieved within 5 mm beyond the tumor margin [28]. The tumor was freeze-thawed using argon and helium gas in a cycle of 15 and 5 min, respectively, and at least 2 cycles were required for each cryoablation. To avoid cold injury to the local skin, 37°C sterilized saline was manually poured on the skin continuously above the frozen tissue throughout the entire process of freezing as described previously [12]. The procedure was completed after the removal of all cryoprobes.

### Follow-up

A single study coordinator (XHY) conducted all follow-up sessions, and CT or MRI findings were evaluated by 5 radiologists (LZL, YW, GST, JYY, and JPL with 15, 14, 8, 30, and 20 years of experience, respectively). Additionally, patients underwent triphasic abdominal CT or MRI and chest radiography 1 month after treatment initiation. Imaging procedures were conducted every 3 months for the first year and every 6 months thereafter. If necessary, positron emission tomography was performed for the diagnosis of metastasis and/or recurrence. The prospective reading was used for imaging data collection. When residual tumor or recurrence was detected, a repeat cryoablation procedure was considered using the same criteria as before. If cryoablation was contraindicated, palliative treatments such as analgesic drug administration were considered. The mRECIST in solid tumors were used to evaluate the local efficacy 1 month after ablation based on acquired images that were evaluated by the 5 radiologists, with findings including complete response (CR), partial response (PR), stable disease (SD), and progressive disease (PD). The response rate (RR) was determined by the sum of the CR and PR rates [29]. The endpoints of interest were progression-free survival (PFS) and overall survival (OS). PFS was defined as the interval between cryoablation and local relapse, distant metastasis, or death, whichever occurred first. OS was calculated as the interval between the date of cryoablation and the date of death from any cause. Adverse events were classified according to the Common Terminology Criteria for Adverse Events v4.0 [30]. The last follow-up date for this study was in June 2015.

### Statistical analyses

All statistical analyses were performed using SPSS for Windows version 18.0 software (SPSS Inc., Chicago, IL). Continuous data, including age, tumor size, and cryoablation probe number were expressed as the mean ± SD and were compared using Student's *t*-test. Categorical data, including adverse events, mRECIST data, invasion, and cryoablation frequency, were presented as frequencies and analyzed using Pearson's χ^2^ test or Fisher's exact test. The PFS and OS curves were constructed using the Kaplan-Meier method and compared using the log-rank test. Prognostic factors predicting PFS and OS were determined using multivariate Cox proportional hazards regression analysis. All statistical tests were two-sided, and differences were considered significant at *P* < 0.05.
